# Salt use patterns and heavy metal urinary excretion

**DOI:** 10.3389/fnut.2024.1521826

**Published:** 2025-01-10

**Authors:** Shuai Zhang, Hanhan Tang, Minglian Zhou, Linqing Pan

**Affiliations:** ^1^Department of Male Reproductive Health, Lianyungang Maternal and Child Health Hospital, Lianyungang, China; ^2^Clinical Center of Reproductive Medicine, Lianyungang Maternal and Child Health Hospital, Lianyungang, China; ^3^Plastic Surgery Department, Xuzhou Central Hospital, Xuzhou, China

**Keywords:** salt, heavy metals, dietary patterns, exposure risk, NHANES

## Abstract

**Background:**

Salt usage patterns have been associated with a risk of multiple diseases; however, their relationship with heavy metal exposure has not been extensively studied.

**Methods:**

This study analyzed survey data from 11,574 NHANES participants. Weighted linear regression models were used to examine the relationship between the type of salt used by participants, the frequency of adding salt at the table, and the frequency of adding regular or seasoned salt to cooking or food preparation, and urinary concentrations of 10 heavy metals. Multiple sensitivity analyses were also performed.

**Results:**

The weighted regression analysis indicated that participants’ salt usage patterns were associated with an increased urinary excretion of certain heavy metals. Specifically, regarding the type of salt used, compared to regular salt, the use of salt substitutes was significantly positively correlated with urinary molybdenum (Mo) levels, while not using salt or substitutes at the table was significantly positively correlated with urinary levels of both Mo and arsenic (As). In terms of the frequency of adding regular salt at the table, frequent addition compared to rarely adding salt was significantly positively correlated with urinary levels of cadmium (Cd), and antimony (Sb), while showing a significant negative correlation with urinary Mo levels. Additionally, when examining the frequency of using regular salt during cooking or food preparation, those who occasionally or very often added regular salt had significantly higher urinary levels of barium (Ba), cesium (Cs), and thallium (Tl) compared to those who never added regular salt during cooking. These associations remained stable in sensitivity analyses.

**Conclusion:**

Our analysis revealed that participants’ salt usage patterns are associated with increased excretion of certain heavy metals, suggesting possible increased exposures to these metals. While these findings are concerning, they require validation in other populations and should be confirmed through prospective studies designed based on this hypothesis.

## 1 Introduction

With industrialization’s progression, the impact of environmental pollutants on human health has become increasingly severe. It is reported that the global disease burden attributed to environmental factors accounts for approximately 8–9% of the total disease burden (1), with even higher proportions observed in developing countries (2). Heavy metals play a particularly significant role in this regard (3). These metals are widely present in the surrounding environment and primarily enter the human body through food, drinking water, inhalation, and skin contact (4). Consuming contaminated food and water is a major source of human heavy metal exposure. Long-term excretion of heavy metals is associated with an increased risk of various diseases, including cardiovascular disease (5), diabetes (6), respiratory diseases (7), dermatological disorders (8), and certain cancers (9). Even at low concentrations, prolonged excretion of heavy metals can adversely affect human health (4, 10). Studies show a significant positive association between excretion of heavy metals and their mixtures and all-cause mortality in U.S. populations (11). In 2019 alone, nearly 1 million deaths globally were attributed to lead (Pb) exposure, accounting for approximately 50% of deaths linked to known chemical exposures (12). This has led to widespread concern regarding heavy metal exposure.

Salt is a common ingredient in food preparation and seasoning; its usage patterns and quantities can significantly impact human health. Adding salt to food is directly linked to an individual’s preference for a salty diet and habitual salt intake (13). The frequency of adding salt can serve as an indicator of long-term preferences for salty flavors and overall sodium intake, which is directly associated with 24-h urinary sodium excretion (14).

Numerous studies have explored the relationship between salt consumption patterns and health outcomes. There is a strong and consistent association between salt intake, consumption of salty foods, and the incidence of gastric cancer and other precancerous conditions, possibly due to heavy metal contamination in salt (15, 16). Previous research has shown that, compared to those who never add salt at the table, individuals who always or frequently do so face an increased risk of gastric, lung, testicular, and bladder cancers. The consumption of processed meats, which generally have high sodium contents, is also significantly associated with an elevated risk of gastrointestinal cancers, urinary system cancers, and malignancies of the lung, prostate, testicles, and blood (17). Research indicates that high sodium intake is strongly associated with increased risks of cardiovascular diseases and related mortality (18). Further studies suggest that the frequency of salt addition to food is closely linked to cardiovascular disease risk, with lower frequencies of salt addition reducing the risk of heart failure and ischemic heart disease (IHD) (14). Moreover, higher frequencies of salt use are significantly associated with increased risks of multiple conditions, including psoriasis (19), chronic kidney disease (20), sleep apnea (21), and type 2 diabetes (22). One study found that individuals who frequently add extra salt to prepared meals have twice the risk of developing type 2 diabetes compared to those who never add salt (23). Furthermore, salt consumption habits have been causally linked to cognitive impairment (24).

In addition to the quantity and frequency of salt used, the type of salt may also influence health outcomes. Recent studies suggest that substituting regular salt with salt alternatives may lower blood pressure and reduce the incidence of stroke, major cardiovascular events, and all-cause mortality (25). However, salt may also pose additional health risks due to contamination with heavy metals (26–29). The cooking process may alter the metal content in food. For instance, Devesa et al. observed a significant increase in arsenic levels in salted cod after cooking (30). Similarly, other studies have reported elevated concentrations of heavy metals—including lead (Pb), cadmium (Cd), arsenic (As), and mercury (Hg)—in salted fish (31). Many researchers attribute the health risks associated with salt consumption primarily to high sodium intake, often overlooking the potential role of contaminant exposure.

Some studies suggest that contaminants in salt may mediate the relationship between salt use and adverse health outcomes (20). This is because some studies have found that the use of 24-h urinary sodium alone may not fully explain the association between the frequency of salt addition and certain health outcomes (32). This raises the question of whether salt usage patterns might influence health outcomes, in part, by altering heavy metal exposure in the body. Could heavy metal exposure act as an intermediary in the complex relationship between salt use and health outcomes? While the effects of salt usage patterns on cardiovascular and other diseases have been widely studied, the relationship between salt consumption patterns and heavy metal exposure remains largely unexplored.

To address these questions and fill this research gap, this study aims to explore the association between salt usage patterns and heavy metal exposure using data from the National Health and Nutrition Examination Survey (NHANES). Specifically, we will examine the relationships between the types of salt regularly used by participants, the frequency of adding salt at the table, and the frequency of adding regular or seasoned salt during cooking or food preparation, with the urinary concentrations of 10 heavy metals: barium (Ba), Cd, cobalt (Co), cesium (Cs), molybdenum (Mo), Pb, antimony (Sb), thallium (Tl), tungsten (W), and As. The findings will provide a deeper understanding of the role of salt usage patterns in certain health outcomes and offer valuable insights into how dietary habits may influence heavy metal exposure. Additionally, this research will provide a crucial reference for future studies investigating the complex interactions among diet, environmental pollutants, and human health.

## 2 Methods

### 2.1 Study population

The data for this study are sourced from NHANES, a cross-sectional survey conducted by the National Center for Health Statistics (NCHS) within the Centers for Disease Control and Prevention (CDC). NHANES is designed to assess the health and nutritional status of U.S. adults and children. Using a complex, multistage, probability sampling design, the survey provides a representative sample of the U.S. population across all age groups. To generate reliable statistical data, NHANES oversampled individuals aged 60 and older, as well as African Americans and Hispanics. The NHANES study protocol was approved by the NCHS Ethics Review Board, and all participants provided informed consent. The NHANES data are fully anonymized public data, collected and utilized in accordance with NHANES privacy protection policies and relevant laws and regulations. All data used in this study are derived from publicly available datasets and do not involve any personally identifiable information; therefore, no additional ethical review is required.

**Figure 1:**
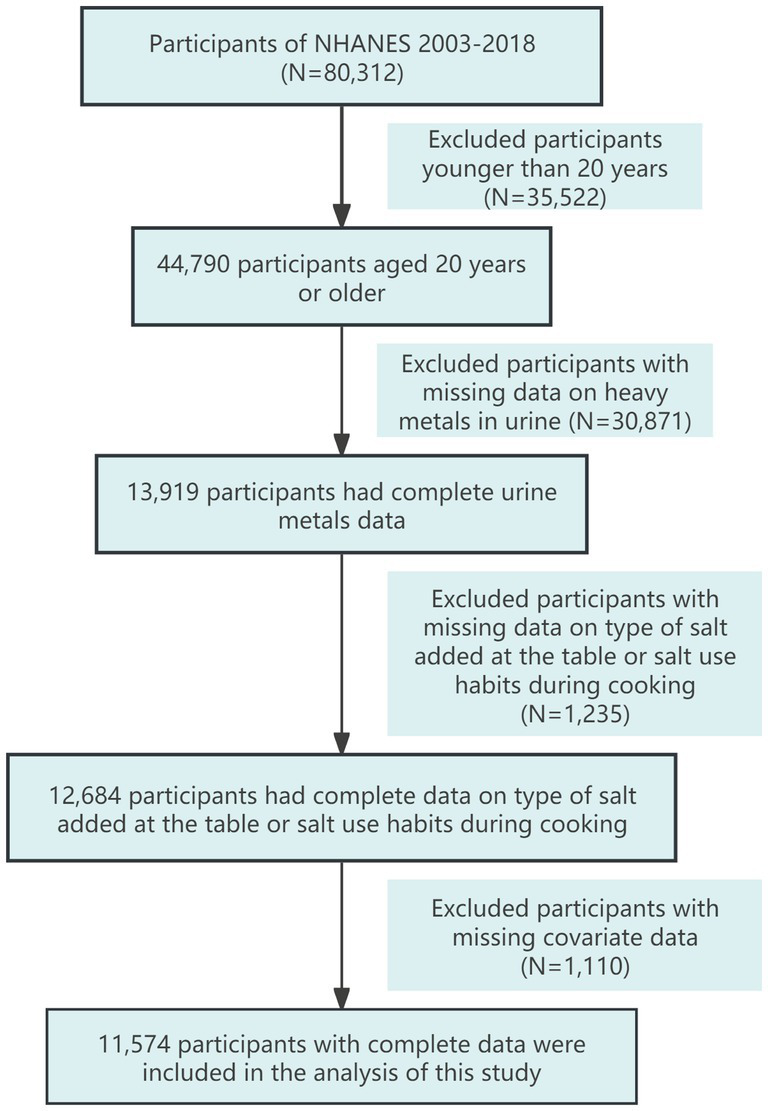
Participant screening flowchart.

Since 2003, NHANES has conducted continuous surveys on salt usage patterns among the U.S. population. Therefore, this study selected participants aged 20 and older from eight consecutive NHANES cycles between 2003 and 2018. We filtered the data by first excluding participants under 20 years old (*N* = 35,522), then excluding those missing urinary metal data (*N* = 30,871), followed by excluding those lacking data on the type of salt added at the table and salt usage habits during cooking (*N* = 1,235). Finally, we excluded participants with missing covariate data (*N* = 1,110), resulting in a total of 11,574 participants included in the analysis. The detailed data filtering process is illustrated in Figure 1.

### 2.2 Heavy metal measurements

Participants were instructed to collect spot urine samples at the Mobile Examination Center (MEC), which were then processed, stored, and transported to the Division of Laboratory Sciences, National Center for Environmental Health, Centers for Disease Control and Prevention in Atlanta, GA, for analysis. All heavy metals were analyzed using inductively coupled plasma dynamic reaction cell mass spectrometry (ICP-DRC-MS). This study included 10 urinary heavy metal elements with detection rates exceeding 76.19%, namely Ba, Cd, Co, Cs, Mo, Pb, Sb, Tl, W, and As, with Sb having the lowest detection rate. For metals with concentrations below the limit of detection (LOD), NHANES replaced the values with LOD divided by the square root of 2. Detailed information on the detection rates of metal elements can be found in Supplementary Table 1. Urinary creatinine levels were also measured to account for urine dilution.

### 2.3 Survey of salt use patterns

Since the 2003–2004 cycle, NHANES has continuously collected data on participants’ salt usage patterns through dietary interviews. During these interviews, participants are asked, “What type of salt do you usually add to your food at the table?” They can choose one answer from four options: 1. Ordinary salt including regular iodized salt, sea salt, and seasoning salts made with regular salt; 2. Lite salt; 3. Salt products or salt substitutes; 4. Does not use or add salt products at the table. So-called lite salt was recorded as such and has a reduced sodium content. Salt substitutes do not contain sodium (33). Low salt is usually a mixture containing NaCl and KCl (34), while salt substitutes usually contain KCl (35). If participants select one of the first three options, they are subsequently asked, “How often do you add ordinary salt to your food at the table?” They can choose one answer from three options: 1. Rarely; 2. Occasionally; 3. Very often. Following this, all participants are asked, “How often is ordinary salt or seasoned salt added in cooking or preparing foods in your household?” They select one answer from four options: 1. Never; 2. Rarely; 3. Occasionally; 4. Very often. Participants who refuse to answer, select “other,” or respond with “I do not know” are assigned a missing value.

### 2.4 Covariates

In this study, several potential confounding factors were considered in the statistical analysis, including demographic characteristics such as age (continuous), gender (male and female), and race/ethnicity (Mexican American, Non-Hispanic White, Non-Hispanic Black, Other Hispanic, and Other groups). Socioeconomic status (SES) indicators included educational attainment (Less than 9th Grade, 9th-11th Grade, High School Graduate/GED or Equivalent, Some College or AA Degree, and College Graduate or Above) and the poverty-to-income ratio (PIR) (≤1, 1–4, and ≥ 4). Additionally, body mass index (BMI) was categorized as underweight (<18.5), normal (18.5 to <25), overweight (25 to <30), and obese (30 or greater), along with smoking status (categorized as never smoker, former smoker, and current smoker).

### 2.5 Statistical analysis

Continuous variables are presented as medians (IQR), while categorical data are expressed as percentages. To minimize the impact of urine dilution, urinary metal concentrations were adjusted using their ratios to urinary creatinine, units in μg/mg creatinine. Since all metal elements exhibited significant skewness, their natural logarithms were used in regression analyses to enhance normality. Taking into account the design of NHANES and following the analysis guidelines published by the National Center for Health Statistics, the two-year metal subsample weights were incorporated in the analysis to ensure that the results are nationally representative.

The primary analysis employed a weighted multivariable linear regression model to examine the association between heavy metal levels and salt usage patterns. The models were adjusted for age, gender, race/ethnicity, educational attainment, PIR, BMI, and smoking status. Results are presented as regression coefficients (*β*) along with their 95% confidence intervals.

### 2.6 Sensitivity analysis

Firstly, as the consumption of fish and shellfish is a significant source of human exposure to certain heavy metals, such as As and Cd (36–38), we further adjusted for participants’ fish and shellfish consumption over the past 30 days in the sensitivity analysis.

Secondly, given that smoking is a significant source of heavy metals in the body (4, 39), we further excluded current smokers and repeated the analysis on the remaining population, adjusting for fish and shellfish intake.

All analyses were conducted using R version 4.4.1, with weighted regression analysis performed using the “survey” package. Our study aims to provide preliminary exploratory insights into the relationship between salt use patterns and heavy metal exposure and to propose potential directions for future prospective research. To maintain sensitivity to the results, no multiple corrections were applied to *p*-values, and a two-sided *p*-value of <0.05 was considered statistically significant (40–43).

## 3 Results

### 3.1 General characteristics of participants and distribution of urinary metals

As shown in the baseline characteristics in Table 1, the median age of participants was 46.0 years (IQR: 33.0, 59.0), with females comprising 52% of the sample. The majority racial/ethnic group was Non-Hispanic White (69%). Most participants had higher educational attainment (Some College or AA degree: 32%) and a high PIR (> 1: 86%), 54% reported never smoking, and 37% were classified as obese.

**Table 1:** Basic characteristics of the study population (*N* = 11,574), NHANES, United States.

	Overall	Type of table salt used				
Characteristic	Overall, *N* = 11,574 (100%)^1^	Ordinary salt, *N* = 7,525 (68%)^1^	Lite salt, *N* = 351 (2.6%)^1^	Salt substitute, *N* = 178 (1.4%)^1^	Does not add salt or substitutes, *N* = 3,520 (28%)^1^	*p*-value^2^
Age (years)	46.0 (33.0, 59.0)	45.0 (32.0, 57.0)	53.0 (33.0, 66.0)	56.0 (38.0, 67.0)	49.0 (35.0, 62.0)	<0.001
Sex						0.027
Female	5,932 (52%)	3,776 (51%)	178 (59%)	91 (55%)	1887 (54%)	
Male	5,642 (48%)	3,749 (49%)	173 (41%)	87 (45%)	1,633 (46%)	
Race/ethnicity						<0.001
Non-Hispanic White	5,228 (69%)	3,539 (71%)	152 (68%)	89 (73%)	1,448 (65%)	
Non-Hispanic Black	2,473 (11%)	1,445 (9.6%)	98 (15%)	50 (15%)	880 (14%)	
Mexican American	1803 (7.9%)	1,264 (8.4%)	42 (6.7%)	21 (6.0%)	476 (6.9%)	
Other/multiracial	1,090 (6.7%)	668 (6.2%)	33 (6.2%)	8 (3.3%)	381 (7.9%)	
Other Hispanic	980 (4.9%)	609 (4.6%)	26 (4.4%)	10 (3.3%)	335 (5.9%)	
Education attainment						0.083
Some College or AA degree	3,460 (32%)	2,249 (32%)	101 (32%)	59 (40%)	1,051 (31%)	
High School Grad/GED	2,706 (24%)	1789 (24%)	98 (27%)	42 (23%)	777 (23%)	
College Graduate or above	2,633 (29%)	1718 (29%)	64 (21%)	37 (22%)	814 (30%)	
9-11th Grade	1,626 (10%)	1,057 (10%)	56 (15%)	23 (11%)	490 (10%)	
Less Than 9th Grade	1,149 (5.2%)	712 (4.9%)	32 (6.2%)	17 (4.2%)	388 (5.9%)	
PIR group						0.6
1 ~ 4	6,253 (50%)	4,029 (50%)	208 (57%)	106 (55%)	1910 (50%)	
≥ 4	2,957 (36%)	1920 (36%)	83 (32%)	38 (33%)	916 (36%)	
≤ 1	2,364 (14%)	1,576 (14%)	60 (12%)	34 (13%)	694 (14%)	
BMI group						0.2
Normal(18.5 to <25)	3,168 (29%)	2,154 (29%)	93 (25%)	41 (23%)	880 (27%)	
Obese(30 or greater)	4,358 (37%)	2,724 (36%)	147 (44%)	84 (44%)	1,403 (38%)	
Overweight(25 to <30)	3,873 (33%)	2,536 (33%)	105 (29%)	52 (32%)	1,180 (33%)	
Underweight(<18.5)	175 (1.4%)	111 (1.4%)	6 (1.9%)	1 (0.8%)	57 (1.4%)	
Smoke group						<0.001
Never smoker	6,313 (54%)	3,944 (52%)	204 (55%)	86 (51%)	2079 (58%)	
Former smoker	2,922 (25%)	1859 (25%)	105 (33%)	62 (34%)	896 (26%)	
Current smoker	2,339 (21%)	1722 (23%)	42 (12%)	30 (15%)	545 (16%)	
Hypertension						<0.001
Hypertensive	6,194 (50%)	3,748 (47%)	208 (59%)	129 (67%)	2,109 (55%)	
Non-Hypertensive	5,380 (50%)	3,777 (53%)	143 (41%)	49 (33%)	1,411 (45%)	
Taking prescription for hypertension						<0.001
Yes	3,509 (26%)	1953 (22%)	146 (42%)	96 (46%)	1,314 (32%)	
No	8,065 (74%)	5,572 (78%)	205 (58%)	82 (54%)	2,206 (68%)	
Ba (μg/L)	1.28 (0.61, 2.45)	1.29 (0.63, 2.48)	1.35 (0.58, 2.05)	1.21 (0.51, 2.34)	1.22 (0.59, 2.43)	0.2
Cd (μg/L)	0.21 (0.10, 0.40)	0.20 (0.09, 0.39)	0.20 (0.11, 0.45)	0.20 (0.12, 0.42)	0.22 (0.11, 0.42)	0.021
Co (μg/L)	0.35 (0.21, 0.56)	0.36 (0.21, 0.56)	0.35 (0.23, 0.49)	0.32 (0.17, 0.53)	0.35 (0.21, 0.55)	0.6
Cs (μg/L)	4.5 (2.6, 6.9)	4.5 (2.6, 6.9)	4.1 (2.4, 6.2)	4.2 (2.2, 7.1)	4.6 (2.7, 6.9)	0.074
Mo (μg/L)	38 (20, 67)	37 (19, 66)	33 (19, 62)	40 (18, 76)	40 (21, 70)	0.033
Pb (μg/L)	0.42 (0.23, 0.75)	0.42 (0.22, 0.75)	0.39 (0.23, 0.72)	0.40 (0.23, 0.73)	0.42 (0.23, 0.75)	0.8
Sb (μg/L)	0.05 (0.03, 0.09)	0.05 (0.03, 0.09)	0.05 (0.03, 0.09)	0.05 (0.02, 0.09)	0.05 (0.03, 0.09)	0.9
Tl (μg/L)	0.16 (0.09, 0.25)	0.16 (0.09, 0.25)	0.13 (0.08, 0.24)	0.13 (0.07, 0.25)	0.16 (0.09, 0.25)	0.054
W (μg/L)	0.06 (0.03, 0.13)	0.06 (0.03, 0.13)	0.06 (0.03, 0.12)	0.06 (0.03, 0.12)	0.07 (0.03, 0.13)	0.6
As (μg/L)	7 (3, 15)	7 (3, 14)	6 (3, 12)	6 (3, 17)	8 (4, 17)	<0.001

1 Median (Q1, Q3); n (unweighted) (%).

2 Design-based KruskalWallis test; Pearson’s X^2: Rao & Scott adjustment.

As shown in Table 1, participants categorized by the type of salt typically added to food exhibit the following age distributions: those using Lite salt and Salt substitute have median ages of 53.0 (33.0, 66.0) and 56.0 (38.0, 67.0), respectively, while participants using Ordinary salt and those who reported not using salt at the table have median ages of 45.0 (32.0, 57.0) and 49.0 (35.0, 62.0), respectively. The proportion of females was significantly higher than that of males across all four types of salt usage (*p* = 0.027). Non-Hispanic Black participants were more likely to use lite salt (15%), salt substitutes (15%), or to avoid adding salt at the table (14%), while Non-Hispanic White participants more commonly used ordinary salt (71%) and salt substitutes (65%). Obese participants were more likely to opt for lite salt (44%) and salt substitutes (44%). Current smokers predominantly chose ordinary salt (23%), possibly influenced by an impaired sense of taste, whereas never-smokers more frequently reported not using table salt (58%).

**Figure 2:**
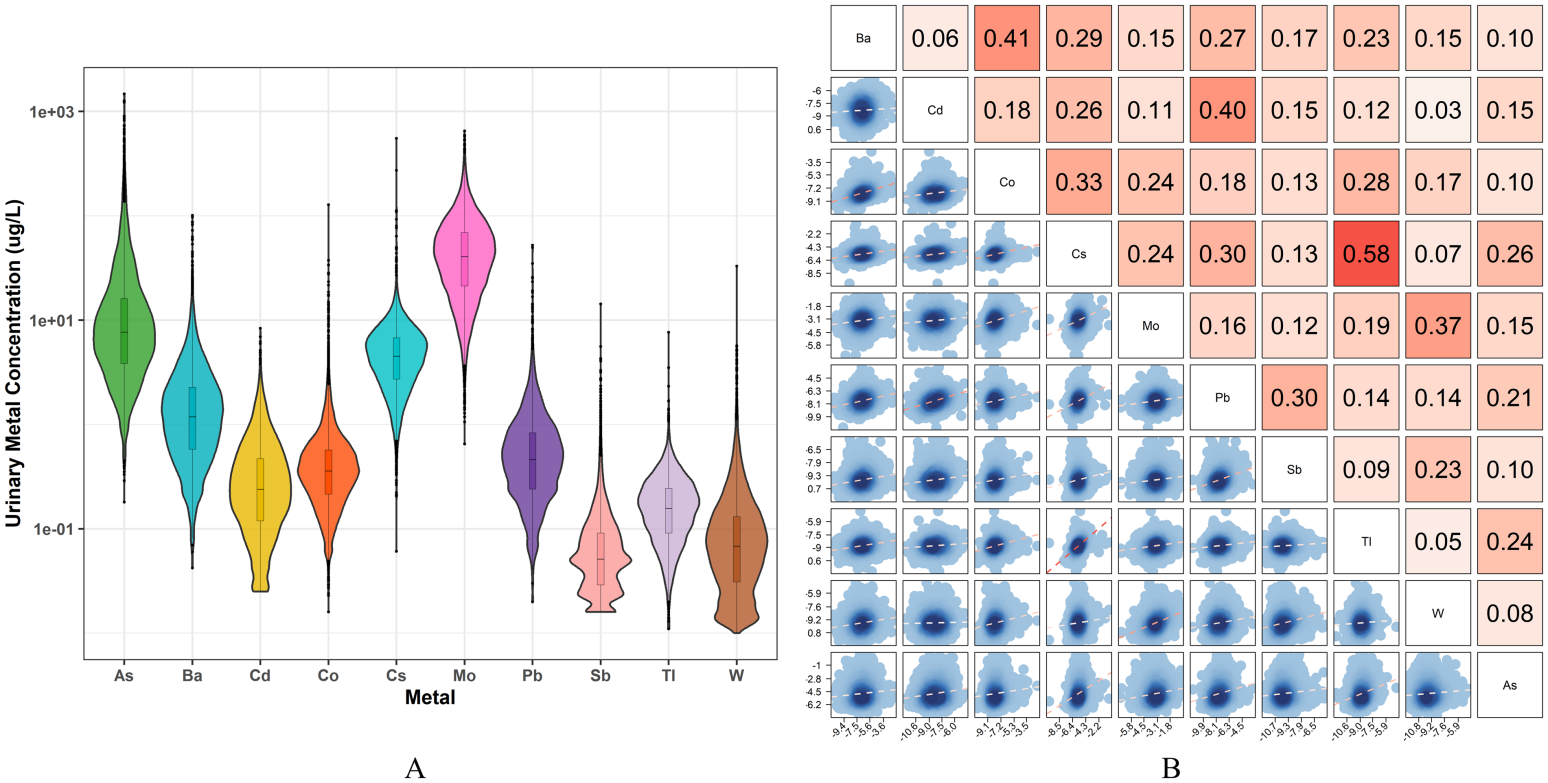
The distribution and correlations of 10 urinary metals. **(A)** Violin plot of urine concentration distribution of 10 heavy metals; **(B)** Pearson correlation analysis of log-transformed urine metals after adjusting for urine dilution, the depth of the red color represents the correlation’s strength.

Figure 2 illustrates the distribution of urinary metals and the results of the correlation analysis. Among the overall participants, the levels of As and Mo are the highest, while the levels of Sb, Tl, and W are the lowest. After adjusting for urine dilution, the Pearson correlation analysis of log-transformed urinary metals revealed significant positive associations among all metals. The strongest correlation was observed between Cs and Tl (*r* = 0.58), followed by Ba and Co (*r* = 0.41) and Cd and Pb (*r* = 0.40).

### 3.2 Urinary metal levels by type of salt used, frequency of adding ordinary salt at the table, and frequency of using ordinary salt during cooking

Table 1 and Supplementary Table 2 present the differences in urinary metal levels categorized by the type of salt used, frequency of adding salt at the table, and frequency of using salt during cooking. As shown in Table 1, the differences in urinary levels of Cd, Mo, and As between the four groups of participants were significant, while the differences in the other metals were not significant.

As shown in Supplementary Table 2, among participants categorized by the frequency of adding salt at the table, those who responded “Very Often” have significantly higher levels of Cd, Pb, and Sb in their urine compared to those who responded “Rarely” and “Occasionally.” For the other metals, the differences among the three groups were not statistically significant. Among the participants divided according to the frequency of salt use during cooking, there were significant differences in urinary levels of Cd, Mo, and Pb among the four groups of participants.

### 3.3 Association between type of salt used, frequency of adding ordinary salt at the table, and frequency of using ordinary salt during cooking with urinary metal levels

The association between urinary metal levels and the type of salt used is presented in Table 2. After adjusting for confounding factors, a correlation was found between the type of salt used and urinary heavy metals. Specifically, the use of salt substitutes was significantly positively correlated with urinary Mo levels compared to the use of ordinary salt, while not using salt or substitutes at the table was significantly positively correlated with urinary Mo and As levels.

**Table 2:** Association between urinary levels of 10 metals and the type of salt used.

Urine metals (μg/mg creatinine)	Type of table salt used	β (95%CI)	*p*-value
Ba	Ordinary salt	Reference	
	Lite salt	0.024(−0.097,0.146)	0.695
	Salt substitute	−0.084(−0.263,0.095)	0.362
	Does not add salt or substitutes	−0.02(−0.066,0.026)	0.401
Cd	Ordinary salt	Reference	
	Lite salt	−0.014(−0.099,0.071)	0.75
	Salt substitute	0.01(−0.105,0.126)	0.86
	Does not add salt or substitutes	0.012(−0.025,0.05)	0.515
Co	Ordinary salt	Reference	
	Lite salt	0.027(−0.05,0.105)	0.491
	Salt substitute	−0.041(−0.153,0.072)	0.479
	Does not add salt or substitutes	−0.023(−0.057,0.012)	0.206
Cs	Ordinary salt	Reference	
	Lite salt	−0.003(−0.094,0.087)	0.946
	Salt substitute	0.002(−0.085,0.09)	0.961
	Does not add salt or substitutes	−0.003(−0.025,0.019)	0.775
Mo	Ordinary salt	Reference	
	Lite salt	0.022(−0.051,0.095)	0.557
	Salt substitute	0.155(0.062,0.247)	0.001
	Does not add salt or substitutes	0.046(0.014,0.078)	0.006
Pb	Ordinary salt	Reference	
	Lite salt	−0.026(−0.117,0.065)	0.578
	Salt substitute	−0.033(−0.169,0.104)	0.639
	Does not add salt or substitutes	−0.016(−0.051,0.019)	0.374
Sb	Ordinary salt	Reference	
	Lite salt	0.063(−0.02,0.146)	0.139
	Salt substitute	0.031(−0.091,0.153)	0.619
	Does not add salt or substitutes	0.002(−0.036,0.04)	0.916
Tl	Ordinary salt	Reference	
	Lite salt	−0.005(−0.084,0.074)	0.896
	Salt substitute	−0.003(−0.11,0.105)	0.96
	Does not add salt or substitutes	0.004(−0.029,0.037)	0.805
W	Ordinary salt	Reference	
	Lite salt	0.074(−0.05,0.198)	0.246
	Salt substitute	0.104(−0.039,0.247)	0.158
	Does not add salt or substitutes	0.04(−0.004,0.083)	0.075
As	Ordinary salt	Reference	
	Lite salt	−0.105(−0.231,0.021)	0.105
	Salt substitute	0.063(−0.101,0.228)	0.452
	Does not add salt or substitutes	0.087(0.031,0.143)	0.003

Barium (Ba), Cadmium (Cd), Cobalt (Co), Cesium (Cs), Molybdenum (Mo), Lead (Pb), Antimony (Sb), Thallium (Tl), Tungsten (W), and Arsenic (As). Models were adjusted for age, gender, race/ethnicity, education attainment, smoking status, PIR, and BMI.

The association between the frequency of adding salt at the table and urinary metal levels is shown in Table 3. Compared to rarely adding salt at the table, frequently adding salt was significantly positively correlated with urinary Cd, Pb, and Sb levels and significantly negatively correlated with urinary Mo levels. Trend analysis indicates a significant positive trend in urinary Cd, Pb, and Sb excretion levels with increasing frequency of table salt use, while urinary Mo levels show a significant negative trend.

**Table 3:** Association between urinary levels of 10 metals and the frequency of adding salt at the table.

Urine metals (μg/mg creatinine)	Frequency of adding salt at the table	β (95%CI)	*p*-value	*p* for trend
Ba	Rarely	Reference		
	Occasionally	0.012(−0.049,0.073)	0.696	0.144
	Very Often	0.045(−0.014,0.103)	0.137	
Cd	Rarely	Reference		
	Occasionally	−0.001(−0.038,0.036)	0.957	0.002
	Very Often	0.086(0.037,0.134)	<0.001	
Co	Rarely	Reference		
	Occasionally	−0.016(−0.053,0.021)	0.403	0.838
	Very Often	0.01(−0.04,0.059)	0.704	
Cs	Rarely	Reference		
	Occasionally	0.003(−0.029,0.035)	0.875	0.286
	Very Often	0.019(−0.013,0.052)	0.245	
Mo	Rarely	Reference		
	Occasionally	−0.034(−0.072,0.004)	0.084	0.014
	Very Often	−0.055(−0.102,-0.009)	0.022	
Pb	Rarely	Reference		
	Occasionally	0.022(−0.021,0.065)	0.316	<0.001
	Very Often	0.156(0.113,0.199)	<0.001	
Sb	Rarely	Reference		
	Occasionally	0.046(0.002,0.091)	0.045	<0.001
	Very Often	0.082(0.036,0.128)	<0.001	
Tl	Rarely	Reference		
	Occasionally	0.011(−0.022,0.043)	0.518	0.745
	Very Often	−0.01(−0.049,0.029)	0.615	
W	Rarely	Reference		
	Occasionally	0.002(−0.055,0.059)	0.944	0.268
	Very Often	−0.047(−0.121,0.028)	0.222	
As	Rarely	Reference		
	Occasionally	−0.006(−0.077,0.064)	0.857	0.367
	Very Often	0.037(−0.03,0.104)	0.285	

Barium (Ba), Cadmium (Cd), Cobalt (Co), Cesium (Cs), Molybdenum (Mo), Lead (Pb), Antimony (Sb), Thallium (Tl), Tungsten (W), and Arsenic (As). Models were adjusted for age, gender, race/ethnicity, education attainment, smoking status, PIR, and BMI.

The association between the frequency of using salt during cooking and urinary metal levels is shown in Table 4. Compared to never adding salt during cooking, occasionally or very often adding salt is significantly positively correlated with urinary levels of Ba, Cs, Pb, and Tl. Trend analysis indicates a significant positive trend in urinary exposure levels of Cs, Pb, Tl, and As with increasing frequency of salt use during cooking, while urinary Mo levels exhibit a significant negative trend.

**Table 4:** Association between urinary levels of 10 metals and the frequency of using salt during cooking or preparing foods.

Urine metals (μg/mg creatinine)	Salt used in preparation	β (95%CI)	*p*-value	*p* for trend
Ba	Never	Reference		
	Rarely	0.047(−0.045,0.139)	0.322	0.071
	Occasionally	0.092(0.009,0.175)	0.032	
	Very Often	0.077(−0.004,0.159)	0.067	
Cd	Never	Reference		
	Rarely	−0.004(−0.068,0.06)	0.907	0.241
	Occasionally	−0.018(−0.078,0.043)	0.569	
	Very Often	0.025(−0.04,0.091)	0.454	
Co	Never	Reference		
	Rarely	−0.01(−0.071,0.052)	0.76	0.114
	Occasionally	−0.005(−0.063,0.052)	0.858	
	Very Often	−0.034(−0.09,0.021)	0.23	
Cs	Never	Reference		
	Rarely	0.076(0.028,0.123)	0.002	<0.001
	Occasionally	0.069(0.024,0.115)	0.003	
	Very Often	0.108(0.07,0.146)	<0.001	
Mo	Never	Reference		
	Rarely	−0.018(−0.09,0.053)	0.622	0.016
	Occasionally	−0.043(−0.109,0.024)	0.211	
	Very Often	−0.063(−0.132,0.006)	0.075	
Pb	Never	Reference		
	Rarely	−0.023(−0.089,0.043)	0.493	<0.001
	Occasionally	0.005(−0.053,0.063)	0.868	
	Very Often	0.075(0.017,0.133)	0.013	
Sb	Never	Reference		
	Rarely	−0.022(−0.095,0.051)	0.554	0.263
	Occasionally	−0.021(−0.093,0.052)	0.578	
	Very Often	−0.04(−0.112,0.032)	0.274	
Tl	Never	Reference		
	Rarely	0.045(−0.009,0.1)	0.106	0.002
	Occasionally	0.057(0.003,0.111)	0.042	
	Very Often	0.083(0.032,0.133)	0.002	
W	Never	Reference		
	Rarely	−0.007(−0.079,0.065)	0.85	0.056
	Occasionally	−0.006(−0.079,0.067)	0.878	
	Very Often	−0.055(−0.124,0.014)	0.122	
As	Never	Reference		
	Rarely	−0.019(−0.117,0.079)	0.705	0.031
	Occasionally	0.008(−0.092,0.108)	0.873	
	Very Often	0.062(−0.043,0.166)	0.25	

Barium (Ba), Cadmium (Cd), Cobalt (Co), Cesium (Cs), Molybdenum (Mo), Lead (Pb), Antimony (Sb), Thallium (Tl), Tungsten (W), and Arsenic (As). Models were adjusted for age, gender, race/ethnicity, education attainment, smoking status, PIR, and BMI.

### 3.4 Sensitivity analysis

After adjusting for fish and shellfish consumption over the past 30 days, the analysis results regarding the association between the type of salt used, the frequency of adding salt at the table, and heavy metals are consistent with the previous findings (Supplementary Tables 3, 4). Compared to never adding salt during cooking, occasionally or very often adding salt is significantly positively correlated with urinary levels of Ba, Cs, Pb, and Tl. Trend analysis indicates a significant positive trend in urinary exposure levels of Cs, Pb, and Tl with increasing frequency of salt use during cooking. Urinary Mo levels continue to exhibit a significant negative trend (Supplementary Table 5).

After excluding current smokers, the analysis results regarding the association between the type of salt used and urinary heavy metals remain consistent with the previous findings (Supplementary Table 6). Compared to rarely adding salt at the table, frequently adding salt is significantly positively correlated with urinary levels of Cd, Pb, and Sb, while it shows a significant negative correlation with urinary Mo levels. Trend analysis indicates a significant positive trend in urinary exposure levels of Pb and Sb with increasing frequency of adding salt at the table. Urinary Mo levels continue to exhibit a significant negative trend (Supplementary Table 7). Compared to never adding salt during cooking, occasionally or very often adding salt is significantly positively correlated with urinary levels of Ba, Cs, and Tl. Trend analysis suggests that with increasing frequency of salt use in cooking, the urinary excretion levels of Cs, Pb, and Tl exhibit a significant positive trend (Supplementary Table 8).

The association between urinary metal levels and the use of antihypertensive prescription medications is shown in Supplementary Table 9. After adjusting for covariates, including age, gender, race/ethnicity, education attainment, smoking status, PIR, BMI, and consumption of shellfish and fish in the past 30 days, the analysis indicates a significant negative correlation between the use of antihypertensive medications and urinary levels of Ba, Cd, Cs, and Pb, while a significant positive correlation was found with urinary W levels. No significant associations were observed between antihypertensive medication use and other metals.

Given the potential impact of antihypertensive medication use, we included the use of antihypertensive drugs as a confounding factor in our sensitivity analysis and conducted a regression analysis to further validate whether the association between salt usage patterns and heavy metal exposure remained stable. The analysis of the association between the type of salt used and urinary metal levels showed that, compared to ordinary salt, the use of salt substitute was significantly positively associated with urinary Mo levels, while not using salt or substitutes at the table was significantly positively associated with urinary Mo and As levels (Supplementary Table 10). In the association between the frequency of salt use at the table and urinary metal levels, frequent use of salt at the table was significantly positively correlated with urinary Cd, Pb, and Sb levels, and negatively correlated with Mo levels, compared to rarely adding salt at the table. Trend tests remained significant (Supplementary Table 11). In the association between salt use during cooking and urinary metal levels, compared to never adding salt during cooking, rare, occasionally, or very often adding salt during cooking was significantly positively associated with urinary Ba, Cs, Pb, and Tl levels (Supplementary Table 12).

## 4 Discussion

To our knowledge, this is the first report on the association between salt usage patterns and heavy metal excretion in the general population. In this cross-sectional study involving 11,574 participants from NHANES, we found that participants’ patterns of salt usage are associated with an increased urinary excretion of certain heavy metals. Specifically, regarding the type of salt used, compared to ordinary salt, which is high in sodium, the use of salt substitutes, which are high in potassium is significantly positively correlated with urinary levels of Mo, while not using salt or substitutes at the table is significantly positively correlated with urinary levels of both Mo and As. Concerning the frequency of adding ordinary salt at the table, frequently adding salt compared to rarely doing so is significantly positively associated with urinary levels of Cd, Pb, and Sb, while being negatively associated with urinary levels of Mo. In terms of the frequency of using ordinary salt during cooking, those who occasionally or very often add salt are significantly positively correlated with urinary levels of Ba, Cs, Pb, and Tl compared to participants who never add salt while cooking.

We found a significant positive correlation between the frequency of adding ordinary salt at the table and/or during cooking and urinary levels of Ba, Cd, Cs, Sb, Pb, and Tl. This association remained stable in sensitivity analyses. We suggest several possible explanations for this correlation: (1) Salt contains trace amounts of heavy metals (44, 45), and with increasing frequency of salt usage, the accumulation of heavy metals in the body may also rise. (2) Salt use may facilitate the release of heavy metal elements from food (46, 47). (3) The dietary habits of these participants may result in higher levels of heavy metals in the food and water they consume. (4) The use of sodium and potassium may influence the absorption and excretion of certain metal elements.

Ba, Cd, Pb, Sb, Tl, and Cs are commonly occurring heavy metal elements that are known to exhibit bioaccumulation and magnification effects (48–52). Some studies have detected the presence of heavy metals in ordinary salt (such as Pb, As, Hg, Cd, and Ba), although the levels are generally considered safe and acceptable (44, 45). Despite significant individual variation in salt intake, the cumulative daily consumption of salt can be substantial (53), potentially contributing to the bioaccumulation and magnification of trace if the salt contains metal contaminants.

The impact of salt use during cooking on the release of heavy metals remains a relatively under-researched area. Although studies have shown that cooking methods and food preparation techniques can influence the bioavailability and release of heavy metals (54, 55), there is a notable lack of in-depth exploration regarding the specific role of salt. Salt serves not only as a seasoning in cooking but may also influence the migration of heavy metals by altering the chemical environment of food and the moisture content. For instance, studies have found that the levels of Hg, Pb, As, and Cd in salted sardines are significantly higher than those in fresh or canned sardines (46). Similarly, salted meat products tend to have relatively high levels of Cs (47). Additionally, some studies have found higher levels of Pb and Cd in certain spices (56). Since salt and spices are often used together, particularly in certain regions or dietary habits, this could further increase the metal burden from dietary intake. Furthermore, changes in pH, salinity, and temperature during cooking may affect the leaching of heavy metals from cooking utensils (57). High temperatures and acidic conditions during cooking can potentially promote the leaching of Tl from aluminum food contact materials, thereby increasing the sources of heavy metal exposure in food (58, 59).

Higher frequencies of salt use at the table and during cooking are associated with increased consumption of processed foods, red meat, and processed meats, along with lower intake of vegetables and fruits (60). More studies have found generally high levels of heavy metals in numerous processed foods (61), including grains, tubers (62, 63), meats (64), and algae (65). Additionally, food packaging materials represent another significant pathway for heavy metal exposure (66, 67). Moreover, higher heavy metal exposure from primary processed foods should not be overlooked (68, 69). Furthermore, excessive salt intake may indirectly lead to increased water consumption (70), and the intake of water contaminated with metals is also a critical pathway for heavy metal exposure.

Our study found that Mo is positively correlated with the use of salt substitutes and not using salt at the table (Table 2), while it shows a negative correlation with the frequency of adding ordinary salt at the table (Table 3) and a significant negative trend with the frequency of using ordinary salt during cooking (Table 4). It is worth noting that in this study, the proportion of participants using salt substitutes was relatively low (1.4%). Despite the limited sample size, we still observed highly significant effects. Currently, there is a lack of reports on the Mo content in salt substitutes. The primary component of salt substitutes is potassium chloride along with some spices and additives. Some studies have found that Mo levels in spices are generally high (71). This may represent an important source of Mo, but further investigation is needed, as the origin and preparation of different spices can significantly affect their Mo content (72). However, participants who tend to choose salt substitutes may primarily follow a lighter vegetarian diet (73), and research indicates that vegetarians have significantly higher Mo intake compared to those on mixed diets (74). This may explain the observed negative trend between the frequency of adding salt at the table and during cooking and Mo excretion. Another possible explanation relates to the types of foods consumed. Participants who frequently add salt during meals and cooking may consume more foods and beverages that are lower in Mo. This demographic often has lower socioeconomic status (75). It has been reported that the main dietary sources of Mo include legumes, grain products, and nuts, with Mo content in legumes ranging from approximately 2.99 to 3.49 μg/g (74). Mo is an essential trace element involved as a cofactor in several key enzymes, such as xanthine oxidase and sulfite oxidase, playing a role in uric acid metabolism and maintaining normal neuronal activity (76). Most Mo intake in the human body comes from food and water (77). Individuals with normal diets and digestive function rarely suffer from Mo deficiency, as sufficient amounts can typically be obtained from food (78). In the body, Mo acts as an antagonist to copper, and excess Mo may lead to various diseases due to copper homeostasis disruption, the interaction of Cu and Mo could similarly mirror an interaction of Na or K and Mo. Additionally, studies have shown that high blood levels of Mo are closely associated with reduced sperm quality in men (79). Regarding hypertension, high airborne Mo exposures are significantly linked to a reduced risk of hypertension (80), and prior research has indicated that using salt substitutes can lower blood pressure (25).

As exposure primarily originates from drinking water, tobacco consumption, and the ingestion of As-contaminated foods (81). Our study found a significant positive correlation between As excretion and not using salt at the table, while no correlation was observed with the frequency of adding ordinary salt at the table or during cooking. This association remained significant even after adjusting for fish and shellfish consumption and excluding smoking participants. It should be noted, however, that the above explanations regarding Mo and As are speculative and currently lack direct supporting evidence. Therefore, targeted studies are needed to further explore and validate these findings.

Whether the use of table salt could influence the absorption and excretion of certain metal elements remains uncertain, as, to the best of our knowledge, no directly relevant studies have been reported. Despite the lack of direct evidence, we propose the following hypothetical mechanisms: (1) Salt intake may affect the intestinal absorption of certain metals. A high-salt diet could potentially damage gastrointestinal epithelial cells or mucosa (82), thereby facilitating the entry of metal ions into the bloodstream. (2) High-salt diets have been found to significantly alter the composition of the gut microbiota (83). Changes in the gut microbiota may, in turn, influence the absorption and excretion of heavy metals by modifying gastrointestinal physiological conditions (e.g., pH), intestinal permeability, and the activity of enzymes involved in heavy metal metabolism (84). (3) The intake levels of sodium and potassium may influence the absorption and excretion of certain metal ions (85–90).

In this study, we analyzed spot urine sample data provided by NHANES to assess metal exposure levels, as it was the only biomarker dataset containing information on multiple metal levels in participants. Urine is one of the most common biomarkers for metal exposure, offering the advantages of being non-invasive and easily obtainable. It has also been widely applied as a marker of human metal exposure in numerous studies (8, 91). However, urinary metal detection does not always provide an accurate or comprehensive reflection of overall metal exposure in the body. Urinary metal levels are closely related to the absorption, biotransformation, and excretion processes of metals within the human body (92). Specifically, urinary Cd is a good biomarker for total body burden and long-term cumulative exposure (93), but urinary Pb is not an effective biomarker for assessing lead exposure in the general population (94). Total As in urine reflects both organic and inorganic As exposure. However, in populations with high fish and seafood intake, urinary total As primarily indicates organic As content rather than the more toxic inorganic As (95). Urinary Ba concentrations mainly reflect recent exposure, typically within the past 3 days to 2 weeks (96). Since Mo is primarily excreted through urine, urinary Mo serves as a good biomarker for short-term Mo exposure, but it is more sensitive to dietary Mo intake (97, 98). Sb primarily enters the body through diet. A study on populations exposed to electronic waste found that occupationally exposed individuals had higher median urinary Sb levels, while blood Sb levels were similar between exposed and unexposed groups (99). Co is also primarily excreted through urine, making urinary Co a critical biomarker for environmental Co exposure (100). Previous studies have shown an association between respiratory and urinary Co levels in workers with occupational Co exposure (101). Cs is excreted approximately 85% through urine, making it a reliable biomarker for Cs exposure (102). Similarly, Tl levels in urine and other biological samples are significantly correlated with environmental Tl exposure (103). For W, its distribution and excretion in mammals are similar to those of Mo, with the majority being filtered by the kidneys and ultimately excreted in urine (104). These findings underscore the objective limitations of using urinary metal elements as biomarkers for human metal exposure. Such limitations pose challenges to the interpretation of our results and highlight the need for caution when inferring associations between salt usage patterns and metal exposure levels based on these findings. Specifically, the associations observed in this study between salt use patterns and Ba, Cd, Cs, Mo, Sb, Tl, and As are more likely to reflect recent or long-term metal exposure levels in participants. However, the association with Pb may not necessarily reflect the participants’ true exposure level.

This study has several unique strengths. First, this study is the first to reveal the potential association between heavy metal urinary excretion and salt consumption patterns. Second, the research assesses various aspects of salt consumption habits, including the type of salt used, the frequency of adding salt at the table, and the frequency of salt use during cooking, in relation to the risk of heavy metal exposure. This study has some limitations. First, the consumption patterns of salt was based on self-reports from participants, which may introduce potential bias. Second, while we adjusted for potential confounding factors, we could not eliminate all influences on heavy metal exposure. Third, the data were sourced from the general population in the United States, and further confirmation is needed regarding their representativeness for other populations. Fourth, due to restricted access to geographic coding data, we were unable to adjust for this information in the regression analyses. However, the potential impacts of dietary patterns and heavy metal exposure levels resulting from geographic differences should not be overlooked. Fifth, the low detection rates for some metal elements may affect statistical power or introduce bias in the results (105, 106). Sixth, this study is based on a cross-sectional design. Although we identified significant associations, whether these associations are causal remains to be further verified.

## 5 Conclusion

In summary, our analysis of 11,574 NHANES participants revealed associations between participants’ salt usage patterns and urinary excretion concentrations of certain heavy metals. Specifically, compared to using regular table salt, which is based on NaCl, the use of salt substitutes, which are typically based on KCl was significantly positively correlated with urinary Mo levels, while the frequency of adding regular salt at the table or during cooking was significantly positively correlated with urinary levels of Ba, Cd, Cs, Sb, and Tl. These findings have uncovered some intriguing interactions between salt use patterns and urinary metal excretion, while also highlighting the complexity of the interplay between dietary habits, metal metabolism, and human health. They also provide public health agencies with critical information that must be taken into account when formulating dietary recommendations concerning salt and salt substitute use. While these findings are potentially concerning, they require validation in other populations and should be confirmed through prospective studies designed based on this hypothesis.

## Data Availability

Publicly available datasets were analyzed in this study. This data can be found at: https://wwwn.cdc.gov/nchs/nhanes/.
