# Supplementary material for: Salt use patterns and heavy metal urinary excretion
Source: Front Nutr. 2025 Jan 10;11:1521826. doi: 10.3389/fnut.2024.1521826 (PMC11757126; doi:10.3389/fnut.2024.1521826)
Supplement: Supplementary file 1 [file Table_1.docx]

**Supplementary Online Content**

**Salt use patterns and heavy metal urinary excretion**

Shuai Zhang ^1,2*#^,Hanhan Tang^3#^ ,Minglian Zhou^1,2^ ,Linqing Pan^2^

**Supplementary Table 1.** The detection rate of urine heavy metal (N=11,574) , NHANES, USA

**Supplementary Table 2.** Urinary metal levels by frequency of adding ordinary salt at the table, and frequency of using ordinary salt during cooking

**Supplementary Table 3.** Association between urinary levels of 10 metals and the type of salt used after further adjustment for fish and shellfish intake

**Supplementary Table 4.** Association between urinary levels of 10 metals and the frequency of adding salt at the table after further adjustment for fish and shellfish intake

**Supplementary Table 5.** Association between urinary levels of 10 metals and the frequency of using salt during cooking or preparing foods after further adjustment for fish and shellfish intake

**Supplementary Table 6.** Association between urinary levels of 10 metals and the type of salt used after excluding current smokers and further adjusting for fish and shellfish intake

**Supplementary Table 7.** Association between urinary levels of 10 metals and the frequency of adding salt at the table after excluding current smokers and further adjusting for fish and shellfish intake

**Supplementary Table 8.** Association between urinary levels of 10 metals and the frequency of using salt during cooking or preparing foods after excluding current smokers and further adjusting for fish and shellfish intake

**Supplementary Table 9.** Association between use of prescribed medications for hypertension and urinary metal levels

**Supplementary Table 10.** Association between urinary levels of 10 metals and the type of salt used after further adjustment for antihypertensive prescription drug use

**Supplementary Table 11.** Association between urinary levels of 10 metals and the frequency of adding salt at the table after further adjustment for antihypertensive prescription drug use

**Supplementary Table 12.** Association between urinary levels of 10 metals and the frequency of using salt during cooking or preparing foods after further adjustment for antihypertensive prescription drug use

**Supplementary Table 1.**The detection rate of urine heavy metal (N=11,574) , NHANES, USA

| **Urine metal** | **LOD (ug/L)** | **Overall detection rate** |
| --- | --- | --- |
| Ba（µg/L） | 0.042 | 98.59% |
| Cd（µg/L） | 0.025 | 83.92% |
| Co（µg/L） | 0.016 | 99.29% |
| Cs（µg/L） | 0.061 | 100% |
| Mo（µg/L） | 0.65 | 99.98% |
| Pb（µg/L） | 0.02 | 96.54% |
| Sb（µg/L） | 0.016 | 76.19% |
| Tl（µg/L） | 0.011 | 99.54% |
| W（µg/L） | 0.01 | 88.60% |
| As（µg/L） | 0.18 | 99.21% |

Note: Barium (Ba), Cadmium (Cd), Cobalt (Co), Cesium (Cs), Molybdenum (Mo), Lead (Pb), Antimony (Sb), Thallium (Tl), Tungsten (W), and Arsenic (As). lower limit of detection(LOD).

**Supplementary Table 2.**Urinary metal levels by frequency of adding ordinary salt at the table, and frequency of using ordinary salt during cooking

|  | **Frequency of adding ordinary salt at the table (N=8046)** | | | | **Ordinary salt used in preparation (N=11574)** | | | | |
| --- | --- | --- | --- | --- | --- | --- | --- | --- | --- |
| **Characteristic** | **Rarely**,  N = 3750 (45%)1 | **Occasionally**,  N = 2428 (32%)1 | **Very Often**,  N = 1868 (23%)1 | **p-value**2 | **Never**,  N = 951 (7.6%)1 | **Rarely**,  N = 2105 (19.5%)1 | **Occasionally**,  N = 3684 (35.4%)1 | **Very Often**,  N = 4834 (37.5%)1 | **p-value**2 |
| **Ba (µg/L)** | 1.27 (0.59, 2.38) | 1.30 (0.63, 2.48) | 1.32 (0.69, 2.53) | 0.3 | 1.14 (0.55, 2.36) | 1.32 (0.64, 2.46) | 1.31 (0.63, 2.55) | 1.24 (0.61, 2.35) | 0.1 |
| **Cd (µg/L)** | 0.19 (0.09, 0.36) | 0.20 (0.09, 0.38) | 0.24 (0.11, 0.47) | <0.001 | 0.24 (0.12, 0.49) | 0.20 (0.10, 0.40) | 0.19 (0.09, 0.37) | 0.21 (0.10, 0.42) | <0.001 |
| **Co (µg/L)** | 0.35 (0.20, 0.57) | 0.36 (0.20, 0.55) | 0.36 (0.22, 0.55) | 0.8 | 0.36 (0.21, 0.58) | 0.36 (0.22, 0.57) | 0.35 (0.21, 0.55) | 0.35 (0.20, 0.55) | 0.14 |
| **Cs (µg/L)** | 4.4 (2.6, 6.8) | 4.4 (2.6, 6.9) | 4.6 (2.7, 7.0) | 0.3 | 4.3 (2.5, 6.5) | 4.5 (2.7, 7.1) | 4.5 (2.6, 6.8) | 4.6 (2.7, 7.0) | 0.2 |
| **Mo (µg/L)** | 38 (19, 67) | 37 (19, 65) | 37 (19, 65) | 0.5 | 40 (22, 69) | 41 (20, 70) | 37 (19, 66) | 37 (20, 66) | 0.047 |
| **Pb (µg/L)** | 0.39 (0.20, 0.70) | 0.40 (0.22, 0.71) | 0.49 (0.25, 0.90) | <0.001 | 0.46 (0.23, 0.85) | 0.40 (0.22, 0.72) | 0.40 (0.22, 0.73) | 0.43 (0.23, 0.76) | 0.002 |
| **Sb (µg/L)** | 0.05 (0.03, 0.09) | 0.05 (0.03, 0.09) | 0.05 (0.03, 0.10) | <0.001 | 0.05 (0.03, 0.09) | 0.05 (0.03, 0.09) | 0.05 (0.03, 0.09) | 0.05 (0.03, 0.09) | 0.2 |
| **Tl (µg/L)** | 0.16 (0.09, 0.25) | 0.16 (0.09, 0.25) | 0.15 (0.09, 0.25) | 0.7 | 0.14 (0.08, 0.25) | 0.16 (0.10, 0.25) | 0.16 (0.09, 0.24) | 0.16 (0.09, 0.26) | 0.091 |
| **W (µg/L)** | 0.06 (0.03, 0.12) | 0.07 (0.03, 0.13) | 0.06 (0.03, 0.12) | 0.9 | 0.07 (0.03, 0.14) | 0.07 (0.03, 0.13) | 0.06 (0.03, 0.13) | 0.06 (0.03, 0.12) | 0.3 |
| **As (µg/L)** | 7 (3, 14) | 6 (3, 13) | 7 (4, 14) | 0.5 | 7 (3, 16) | 7 (3, 14) | 7 (3, 14) | 7 (4, 16) | 0.12 |

^1^Median (Q1, Q3); n (unweighted) (%).

^2^Design-based KruskalWallis test; Pearson's X^2: Rao & Scott adjustment.

Note: Barium (Ba), Cadmium (Cd), Cobalt (Co), Cesium (Cs), Molybdenum (Mo), Lead (Pb), Antimony (Sb), Thallium (Tl), Tungsten (W), and Arsenic (As).

**Supplementary Table 3.** Association between urinary levels of 10 metals and the type of salt used after further adjustment for fish and shellfish intake

| **Urine metals**  **(µg/mg creatinine)** | **Type of table salt used** | **β (95%CI)** | **p-value** |
| --- | --- | --- | --- |
| Ba | Ordinary salt | Reference |  |
|  | Lite salt | 0.017(-0.108,0.141) | 0.795 |
|  | Salt substitute | -0.058(-0.255,0.139) | 0.564 |
|  | Doesn't add salt or substitutes | -0.02(-0.069,0.029) | 0.415 |
| Cd | Ordinary salt | Reference |  |
|  | Lite salt | -0.022(-0.108,0.064) | 0.623 |
|  | Salt substitute | 0.015(-0.11,0.14) | 0.813 |
|  | Doesn't add salt or substitutes | 0.012(-0.026,0.05) | 0.531 |
| Co | Ordinary salt | Reference |  |
|  | Lite salt | 0.025(-0.056,0.106) | 0.551 |
|  | Salt substitute | -0.025(-0.143,0.094) | 0.685 |
|  | Doesn't add salt or substitutes | -0.019(-0.055,0.018) | 0.317 |
| Cs | Ordinary salt | Reference |  |
|  | Lite salt | 0.003(-0.094,0.1) | 0.953 |
|  | Salt substitute | -0.005(-0.098,0.087) | 0.908 |
|  | Doesn't add salt or substitutes | 0(-0.024,0.024) | 1 |
| Mo | Ordinary salt | Reference |  |
|  | Lite salt | 0.037(-0.038,0.112) | 0.337 |
|  | Salt substitute | 0.166(0.067,0.265) | 0.001 |
|  | Doesn't add salt or substitutes | 0.053(0.02,0.087) | 0.002 |
| Pb | Ordinary salt | Reference |  |
|  | Lite salt | -0.019(-0.113,0.075) | 0.698 |
|  | Salt substitute | -0.039(-0.184,0.105) | 0.595 |
|  | Doesn't add salt or substitutes | -0.024(-0.059,0.011) | 0.18 |
| Sb | Ordinary salt | Reference |  |
|  | Lite salt | 0.064(-0.024,0.152) | 0.158 |
|  | Salt substitute | 0.028(-0.093,0.149) | 0.65 |
|  | Doesn't add salt or substitutes | -0.008(-0.044,0.029) | 0.674 |
| Tl | Ordinary salt | Reference |  |
|  | Lite salt | -0.014(-0.096,0.068) | 0.738 |
|  | Salt substitute | 0.005(-0.108,0.119) | 0.927 |
|  | Doesn't add salt or substitutes | 0.005(-0.028,0.039) | 0.751 |
| W | Ordinary salt | Reference |  |
|  | Lite salt | 0.085(-0.043,0.214) | 0.196 |
|  | Salt substitute | 0.109(-0.046,0.263) | 0.171 |
|  | Doesn't add salt or substitutes | 0.042(-0.004,0.088) | 0.079 |
| As | Ordinary salt | Reference |  |
|  | Lite salt | -0.065(-0.2,0.069) | 0.344 |
|  | Salt substitute | 0.044(-0.105,0.193) | 0.565 |
|  | Doesn't add salt or substitutes | 0.096(0.039,0.152) | 0.001 |

Note: Barium (Ba), Cadmium (Cd), Cobalt (Co), Cesium (Cs), Molybdenum (Mo), Lead (Pb), Antimony (Sb), Thallium (Tl), Tungsten (W), and Arsenic (As).

Models were adjusted for age, gender, race/ethnicity, education attainment, smoking status, PIR, BMI and consumption of shellfish and fish in the past 30 days.

**Supplementary Table 4.** Association between urinary levels of 10 metals and the frequency of adding salt at the table after further adjustment for fish and shellfish intake

| **Urine metals (µg/mg creatinine)** | **Frequency of adding salt at the table** | **β (95%CI)** | **p-value** | **p for trend** |
| --- | --- | --- | --- | --- |
| Ba | Rarely | Reference |  |  |
|  | Occasionally | 0.014(-0.049,0.077) | 0.67 | 0.21 |
|  | Very Often | 0.041(-0.023,0.106) | 0.212 |  |
| Cd | Rarely | Reference |  |  |
|  | Occasionally | -0.004(-0.042,0.034) | 0.831 | 0.029 |
|  | Very Often | 0.065(0.012,0.119) | 0.018 |  |
| Co | Rarely | Reference |  |  |
|  | Occasionally | -0.009(-0.048,0.029) | 0.639 | 0.426 |
|  | Very Often | 0.025(-0.027,0.078) | 0.341 |  |
| Cs | Rarely | Reference |  |  |
|  | Occasionally | -0.003(-0.036,0.029) | 0.85 | 0.708 |
|  | Very Often | 0.008(-0.026,0.043) | 0.633 |  |
| Mo | Rarely | Reference |  |  |
|  | Occasionally | -0.042(-0.081,-0.004) | 0.033 | 0.002 |
|  | Very Often | -0.071(-0.116,-0.025) | 0.003 |  |
| Pb | Rarely | Reference |  |  |
|  | Occasionally | 0.024(-0.018,0.066) | 0.271 | <0.001 |
|  | Very Often | 0.137(0.091,0.182) | <0.001 |  |
| Sb | Rarely | Reference |  |  |
|  | Occasionally | 0.045(0.001,0.089) | 0.049 | 0.005 |
|  | Very Often | 0.066(0.017,0.115) | 0.01 |  |
| Tl | Rarely | Reference |  |  |
|  | Occasionally | 0.013(-0.02,0.046) | 0.441 | 0.803 |
|  | Very Often | -0.01(-0.051,0.032) | 0.651 |  |
| W | Rarely | Reference |  |  |
|  | Occasionally | -0.002(-0.062,0.058) | 0.951 | 0.249 |
|  | Very Often | -0.048(-0.123,0.026) | 0.205 |  |
| As | Rarely | Reference |  |  |
|  | Occasionally | 0.001(-0.066,0.067) | 0.981 | 0.255 |
|  | Very Often | 0.044(-0.023,0.11) | 0.199 |  |

Note: Barium (Ba), Cadmium (Cd), Cobalt (Co), Cesium (Cs), Molybdenum (Mo), Lead (Pb), Antimony (Sb), Thallium (Tl), Tungsten (W), and Arsenic (As).

Models were adjusted for age, gender, race/ethnicity, education attainment, smoking status, PIR, BMI and consumption of shellfish and fish in the past 30 days.

**Supplementary Table 5.** Association between urinary levels of 10 metals and the frequency of using salt during cooking or preparing foods after further adjustment for fish and shellfish intake

| **Urine metals (µg/mg creatinine)** | **Salt used in preparation** | **β (95%CI)** | **p-value** | **p for trend** |
| --- | --- | --- | --- | --- |
| Ba | Never | Reference |  |  |
|  | Rarely | 0.047(-0.043,0.137) | 0.312 | 0.21 |
|  | Occasionally | 0.098(0.016,0.18) | 0.022 |  |
|  | Very Often | 0.065(-0.019,0.149) | 0.133 |  |
| Cd | Never | Reference |  |  |
|  | Rarely | 0.001(-0.065,0.067) | 0.973 | 0.307 |
|  | Occasionally | -0.013(-0.076,0.05) | 0.69 |  |
|  | Very Often | 0.024(-0.04,0.087) | 0.462 |  |
| Co | Never | Reference |  |  |
|  | Rarely | -0.013(-0.08,0.055) | 0.717 | 0.262 |
|  | Occasionally | -0.003(-0.066,0.06) | 0.928 |  |
|  | Very Often | -0.03(-0.091,0.032) | 0.349 |  |
| Cs | Never | Reference |  |  |
|  | Rarely | 0.071(0.021,0.121) | 0.006 | <0.001 |
|  | Occasionally | 0.065(0.017,0.112) | 0.009 |  |
|  | Very Often | 0.1(0.06,0.14) | <0.001 |  |
| Mo | Never | Reference |  |  |
|  | Rarely | -0.004(-0.079,0.07) | 0.912 | 0.029 |
|  | Occasionally | -0.019(-0.088,0.05) | 0.583 |  |
|  | Very Often | -0.051(-0.123,0.021) | 0.166 |  |
| Pb | Never | Reference |  |  |
|  | Rarely | -0.03(-0.101,0.041) | 0.405 | <0.001 |
|  | Occasionally | 0.009(-0.051,0.07) | 0.759 |  |
|  | Very Often | 0.062(0,0.124) | 0.05 |  |
| Sb | Never | Reference |  |  |
|  | Rarely | -0.019(-0.096,0.059) | 0.641 | 0.189 |
|  | Occasionally | -0.009(-0.082,0.063) | 0.799 |  |
|  | Very Often | -0.043(-0.118,0.031) | 0.256 |  |
| Tl | Never | Reference |  |  |
|  | Rarely | 0.042(-0.015,0.098) | 0.149 | <0.001 |
|  | Occasionally | 0.054(0.001,0.108) | 0.05 |  |
|  | Very Often | 0.083(0.035,0.131) | 0.001 |  |
| W | Never | Reference |  |  |
|  | Rarely | -0.012(-0.089,0.065) | 0.766 | 0.06 |
|  | Occasionally | 0.003(-0.076,0.082) | 0.939 |  |
|  | Very Often | -0.058(-0.13,0.014) | 0.115 |  |
| As | Never | Reference |  |  |
|  | Rarely | -0.041(-0.141,0.058) | 0.419 | 0.3 |
|  | Occasionally | -0.025(-0.127,0.076) | 0.627 |  |
|  | Very Often | 0.009(-0.096,0.114) | 0.865 |  |

Note: Barium (Ba), Cadmium (Cd), Cobalt (Co), Cesium (Cs), Molybdenum (Mo), Lead (Pb), Antimony (Sb), Thallium (Tl), Tungsten (W), and Arsenic (As).

Models were adjusted for age, gender, race/ethnicity, education attainment, smoking status, PIR, BMI and consumption of shellfish and fish in the past 30 days.

**Supplementary Table 6.** Association between urinary levels of 10 metals and type of salt used after excluding current smokers and further adjusting for fish and shellfish intake

| **Urine metals (µg/mg creatinine)** | **Type of table salt used** | **β (95%CI)** | **p-value** |
| --- | --- | --- | --- |
| Ba | Ordinary salt | Reference |  |
|  | Lite salt | 0.021(-0.106,0.147) | 0.751 |
|  | Salt substitute | -0.019(-0.23,0.193) | 0.863 |
|  | Doesn't add salt or substitutes | -0.025(-0.081,0.032) | 0.396 |
| Cd | Ordinary salt | Reference |  |
|  | Lite salt | -0.012(-0.107,0.082) | 0.798 |
|  | Salt substitute | 0.08(-0.056,0.215) | 0.252 |
|  | Doesn't add salt or substitutes | 0.01(-0.032,0.052) | 0.644 |
| Co | Ordinary salt | Reference |  |
|  | Lite salt | 0.055(-0.035,0.145) | 0.237 |
|  | Salt substitute | -0.022(-0.14,0.097) | 0.72 |
|  | Doesn't add salt or substitutes | -0.015(-0.059,0.029) | 0.503 |
| Cs | Ordinary salt | Reference |  |
|  | Lite salt | -0.011(-0.096,0.074) | 0.808 |
|  | Salt substitute | 0.02(-0.081,0.121) | 0.701 |
|  | Doesn't add salt or substitutes | 0.003(-0.023,0.029) | 0.814 |
| Mo | Ordinary salt | Reference |  |
|  | Lite salt | 0.043(-0.039,0.125) | 0.304 |
|  | Salt substitute | 0.202(0.094,0.31) | <0.001 |
|  | Doesn't add salt or substitutes | 0.05(0.017,0.084) | 0.004 |
| Pb | Ordinary salt | Reference |  |
|  | Lite salt | 0.002(-0.099,0.104) | 0.964 |
|  | Salt substitute | 0.006(-0.145,0.157) | 0.933 |
|  | Doesn't add salt or substitutes | -0.028(-0.066,0.011) | 0.161 |
| Sb | Ordinary salt | Reference |  |
|  | Lite salt | 0.074(-0.02,0.169) | 0.127 |
|  | Salt substitute | 0.046(-0.076,0.168) | 0.461 |
|  | Doesn't add salt or substitutes | -0.017(-0.057,0.023) | 0.401 |
| Tl | Ordinary salt | Reference |  |
|  | Lite salt | -0.01(-0.093,0.074) | 0.824 |
|  | Salt substitute | 0.029(-0.095,0.153) | 0.645 |
|  | Doesn't add salt or substitutes | 0.012(-0.025,0.05) | 0.526 |
| W | Ordinary salt | Reference |  |
|  | Lite salt | 0.087(-0.022,0.196) | 0.12 |
|  | Salt substitute | 0.083(-0.087,0.254) | 0.339 |
|  | Doesn't add salt or substitutes | 0.029(-0.023,0.081) | 0.28 |
| As | Ordinary salt | Reference |  |
|  | Lite salt | -0.082(-0.227,0.063) | 0.271 |
|  | Salt substitute | 0.064(-0.11,0.238) | 0.473 |
|  | Doesn't add salt or substitutes | 0.101(0.039,0.162) | 0.002 |

Note: Barium (Ba), Cadmium (Cd), Cobalt (Co), Cesium (Cs), Molybdenum (Mo), Lead (Pb), Antimony (Sb), Thallium (Tl), Tungsten (W), and Arsenic (As).

Models were adjusted for age, gender, race/ethnicity, education attainment, smoking status, PIR, BMI and consumption of shellfish and fish in the past 30 days.

**Supplementary Table 7.** Association between urinary levels of 10 metals and the frequency of adding salt at the table after excluding current smokers and further adjusting for fish and shellfish intake

| **Urine metals (µg/mg creatinine)** | **Frequency of adding salt at the table** | **β (95%CI)** | **p-value** | **p for trend** |
| --- | --- | --- | --- | --- |
| Ba | Rarely | Reference |  |  |
|  | Occasionally | 0.053(-0.022,0.129) | 0.168 | 0.078 |
|  | Very Often | 0.052(-0.016,0.12) | 0.14 |  |
| Cd | Rarely | Reference |  |  |
|  | Occasionally | -0.004(-0.046,0.038) | 0.852 | 0.055 |
|  | Very Often | 0.058(0.007,0.109) | 0.027 |  |
| Co | Rarely | Reference |  |  |
|  | Occasionally | -0.013(-0.061,0.034) | 0.585 | 0.923 |
|  | Very Often | 0.001(-0.054,0.057) | 0.959 |  |
| Cs | Rarely | Reference |  |  |
|  | Occasionally | -0.003(-0.041,0.036) | 0.896 | 0.949 |
|  | Very Often | 0.002(-0.037,0.042) | 0.908 |  |
| Mo | Rarely | Reference |  |  |
|  | Occasionally | -0.048(-0.092,-0.004) | 0.036 | 0.002 |
|  | Very Often | -0.082(-0.136,-0.028) | 0.004 |  |
| Pb | Rarely | Reference |  |  |
|  | Occasionally | 0.032(-0.016,0.081) | 0.191 | <0.001 |
|  | Very Often | 0.138(0.086,0.191) | <0.001 |  |
| Sb | Rarely | Reference |  |  |
|  | Occasionally | 0.059(0.007,0.111) | 0.027 | 0.002 |
|  | Very Often | 0.082(0.026,0.137) | 0.005 |  |
| Tl | Rarely | Reference |  |  |
|  | Occasionally | 0.016(-0.022,0.054) | 0.405 | 0.483 |
|  | Very Often | -0.025(-0.074,0.023) | 0.307 |  |
| W | Rarely | Reference |  |  |
|  | Occasionally | -0.002(-0.066,0.061) | 0.939 | 0.319 |
|  | Very Often | -0.049(-0.135,0.038) | 0.27 |  |
| As | Rarely | Reference |  |  |
|  | Occasionally | 0.01(-0.068,0.087) | 0.807 | 0.195 |
|  | Very Often | 0.058(-0.022,0.139) | 0.156 |  |

Note: Barium (Ba), Cadmium (Cd), Cobalt (Co), Cesium (Cs), Molybdenum (Mo), Lead (Pb), Antimony (Sb), Thallium (Tl), Tungsten (W), and Arsenic (As).

Models were adjusted for age, gender, race/ethnicity, education attainment, smoking status, PIR, BMI and consumption of shellfish and fish in the past 30 days.

**Supplementary Table 8.** Association between urinary levels of 10 metals and the frequency of using salt during cooking or preparing foods after excluding current smokers and further adjusting for fish and shellfish intake

| **Urine metals (µg/mg creatinine)** | **Salt used in preparation** | **β (95%CI)** | **p-value** | **p for trend** |
| --- | --- | --- | --- | --- |
| Ba | Never | Reference |  |  |
|  | Rarely | 0.072(-0.028,0.171) | 0.162 | 0.199 |
|  | Occasionally | **0.114(0.02,0.208)** | **0.02** |  |
|  | Very Often | **0.085(-0.01,0.179)** | **0.083** |  |
| Cd | Never | Reference |  |  |
|  | Rarely | 0.012(-0.06,0.085) | 0.741 | 0.166 |
|  | Occasionally | 0.005(-0.063,0.072) | 0.891 |  |
|  | Very Often | 0.04(-0.026,0.106) | 0.234 |  |
| Co | Never | Reference |  |  |
|  | Rarely | 0.006(-0.069,0.082) | 0.87 | 0.361 |
|  | Occasionally | 0.012(-0.059,0.084) | 0.731 |  |
|  | Very Often | -0.018(-0.089,0.053) | 0.614 |  |
| Cs | Never | Reference |  |  |
|  | Rarely | 0.074(0.022,0.127) | 0.006 | <0.001 |
|  | Occasionally | 0.065(0.014,0.116) | 0.015 |  |
|  | Very Often | 0.108(0.062,0.154) | <0.001 |  |
| Mo | Never | Reference |  |  |
|  | Rarely | 0.016(-0.059,0.09) | 0.681 | 0.058 |
|  | Occasionally | -0.016(-0.087,0.055) | 0.657 |  |
|  | Very Often | -0.043(-0.119,0.033) | 0.272 |  |
| Pb | Never | Reference |  |  |
|  | Rarely | -0.043(-0.119,0.032) | 0.264 | 0.007 |
|  | Occasionally | -0.009(-0.077,0.06) | 0.804 |  |
|  | Very Often | 0.047(-0.022,0.115) | 0.187 |  |
| Sb | Never | Reference |  |  |
|  | Rarely | -0.026(-0.114,0.063) | 0.572 | 0.142 |
|  | Occasionally | -0.013(-0.095,0.068) | 0.751 |  |
|  | Very Often | -0.056(-0.137,0.026) | 0.184 |  |
| Tl | Never | Reference |  |  |
|  | Rarely | 0.053(-0.014,0.12) | 0.121 | 0.002 |
|  | Occasionally | 0.055(-0.01,0.12) | 0.102 |  |
|  | Very Often | 0.092(0.035,0.15) | 0.002 |  |
| W | Never | Reference |  |  |
|  | Rarely | -0.009(-0.094,0.077) | 0.841 | 0.131 |
|  | Occasionally | 0.007(-0.081,0.095) | 0.883 |  |
|  | Very Often | -0.055(-0.139,0.028) | 0.199 |  |
| As | Never | Reference |  |  |
|  | Rarely | -0.039(-0.15,0.073) | 0.498 | 0.459 |
|  | Occasionally | -0.033(-0.14,0.073) | 0.541 |  |
|  | Very Often | 0.005(-0.11,0.119) | 0.939 |  |

Note: Barium (Ba), Cadmium (Cd), Cobalt (Co), Cesium (Cs), Molybdenum (Mo), Lead (Pb), Antimony (Sb), Thallium (Tl), Tungsten (W), and Arsenic (As).

Models were adjusted for age, gender, race/ethnicity, education attainment, smoking status, PIR, BMI and consumption of shellfish and fish in the past 30 days.

**Supplementary Table 9. Association between use of prescribed medications for hypertension and urinary metal levels**

| **Urine metals (µg/mg creatinine)** | **Taking prescription for hypertension** | **β (95%CI)** | **p-value** |
| --- | --- | --- | --- |
| Ba | No | Reference |  |
|  | Yes | -0.207(-0.257,-0.156) | < 0.001 |
| Cd | No | Reference |  |
|  | Yes | -0.041(-0.08,-0.003) | 0.037 |
| Co | No | Reference |  |
|  | Yes | -0.023(-0.064,0.018) | 0.271 |
| Cs | No | Reference |  |
|  | Yes | -0.06(-0.087,-0.032) | < 0.001 |
| Mo | No | Reference |  |
|  | Yes | 0.033(-0.007,0.074) | 0.111 |
| Pb | No | Reference |  |
|  | Yes | -0.138(-0.175,-0.101) | < 0.001 |
| Sb | No | Reference |  |
|  | Yes | 0.01(-0.033,0.053) | 0.635 |
| Tl | No | Reference |  |
|  | Yes | -0.035(-0.071,0.001) | 0.062 |
| W | No | Reference |  |
|  | Yes | 0.084(0.029,0.14) | 0.004 |
| As | No | Reference |  |
|  | Yes | 0.058(-0.002,0.119) | 0.063 |

Note: Models were adjusted for age, gender, race/ethnicity, education attainment, smoking status, PIR, BMI, and consumption of shellfish and fish in the past 30 days.

**Supplementary Table 10. Association between urinary levels of 10 metals and the type of salt used after further adjustment for antihypertensive prescription drug use**

| **Urine metals (µg/mg creatinine)** | **Type of table salt used** | **β (95%CI)** | **p-value** |
| --- | --- | --- | --- |
| Ba | Ordinary salt | Reference |  |
|  | Lite salt | 0.047(-0.074,0.168) | 0.447 |
|  | Salt substitute | -0.06(-0.24,0.119) | 0.511 |
|  | Doesn't add salt or substitutes | -0.01(-0.056,0.036) | 0.663 |
| Cd | Ordinary salt | Reference |  |
|  | Lite salt | -0.009(-0.095,0.076) | 0.829 |
|  | Salt substitute | 0.015(-0.101,0.131) | 0.801 |
|  | Doesn't add salt or substitutes | 0.014(-0.023,0.052) | 0.459 |
| Co | Ordinary salt | Reference |  |
|  | Lite salt | 0.03(-0.046,0.107) | 0.443 |
|  | Salt substitute | -0.038(-0.15,0.074) | 0.51 |
|  | Doesn't add salt or substitutes | -0.021(-0.056,0.013) | 0.228 |
| Cs | Ordinary salt | Reference |  |
|  | Lite salt | 0.004(-0.087,0.096) | 0.931 |
|  | Salt substitute | 0.01(-0.078,0.097) | 0.83 |
|  | Doesn't add salt or substitutes | 0(-0.022,0.021) | 0.99 |
| Mo | Ordinary salt | Reference |  |
|  | Lite salt | 0.02(-0.053,0.094) | 0.589 |
|  | Salt substitute | 0.153(0.06,0.246) | 0.002 |
|  | Doesn't add salt or substitutes | 0.045(0.013,0.078) | 0.007 |
| Pb | Ordinary salt | Reference |  |
|  | Lite salt | -0.01(-0.103,0.082) | 0.826 |
|  | Salt substitute | -0.017(-0.152,0.119) | 0.809 |
|  | Doesn't add salt or substitutes | -0.009(-0.044,0.026) | 0.602 |
| Sb | Ordinary salt | Reference |  |
|  | Lite salt | 0.062(-0.022,0.146) | 0.151 |
|  | Salt substitute | 0.03(-0.092,0.152) | 0.634 |
|  | Doesn't add salt or substitutes | 0.002(-0.037,0.04) | 0.937 |
| Tl | Ordinary salt | Reference |  |
|  | Lite salt | 0(-0.079,0.08) | 0.995 |
|  | Salt substitute | 0.003(-0.105,0.111) | 0.958 |
|  | Doesn't add salt or substitutes | 0.007(-0.027,0.04) | 0.7 |
| W | Ordinary salt | Reference |  |
|  | Lite salt | 0.066(-0.058,0.19) | 0.302 |
|  | Salt substitute | 0.096(-0.048,0.239) | 0.194 |
|  | Doesn't add salt or substitutes | 0.036(-0.007,0.079) | 0.102 |
| As | Ordinary salt | Reference |  |
|  | Lite salt | -0.111(-0.238,0.016) | 0.09 |
|  | Salt substitute | 0.057(-0.108,0.223) | 0.499 |
|  | Doesn't add salt or substitutes | 0.085(0.028,0.141) | 0.004 |

Note: Models were adjusted for age, gender, race/ethnicity, education attainment, smoking status, PIR, BMI, consumption of shellfish and fish in the past 30 days, and antihypertensive prescription drug use.

**Supplementary Table 11. Association between urinary levels of 10 metals and the frequency of adding salt at the table after further adjustment for antihypertensive prescription drug use**

| **Urine metals (µg/mg creatinine)** | **Frequency of adding salt at the table** | **β (95%CI)** | **p-value** | **p for trend** |
| --- | --- | --- | --- | --- |
| Ba | Rarely | Reference |  |  |
|  | Occasionally | 0.01(-0.05,0.069) | 0.753 | 0.213 |
|  | Very Often | 0.038(-0.02,0.097) | 0.204 |  |
| Cd | Rarely | Reference |  |  |
|  | Occasionally | -0.002(-0.038,0.035) | 0.936 | 0.002 |
|  | Very Often | 0.084(0.035,0.134) | 0.001 |  |
| Co | Rarely | Reference |  |  |
|  | Occasionally | -0.016(-0.054,0.021) | 0.392 | 0.875 |
|  | Very Often | 0.009(-0.041,0.058) | 0.738 |  |
| Cs | Rarely | Reference |  |  |
|  | Occasionally | 0.002(-0.03,0.034) | 0.919 | 0.353 |
|  | Very Often | 0.017(-0.016,0.05) | 0.307 |  |
| Mo | Rarely | Reference |  |  |
|  | Occasionally | -0.034(-0.072,0.004) | 0.085 | 0.015 |
|  | Very Often | -0.055(-0.101,-0.008) | 0.023 |  |
| Pb | Rarely | Reference |  |  |
|  | Occasionally | 0.02(-0.022,0.063) | 0.355 | <0.001 |
|  | Very Often | 0.151(0.109,0.194) | <0.001 |  |
| Sb | Rarely | Reference |  |  |
|  | Occasionally | 0.046(0.002,0.091) | 0.044 | <0.001 |
|  | Very Often | 0.082(0.036,0.128) | 0.001 |  |
| Tl | Rarely | Reference |  |  |
|  | Occasionally | 0.01(-0.022,0.042) | 0.548 | 0.673 |
|  | Very Often | -0.012(-0.051,0.027) | 0.548 |  |
| W | Rarely | Reference |  |  |
|  | Occasionally | 0.003(-0.054,0.059) | 0.928 | 0.286 |
|  | Very Often | -0.045(-0.119,0.029) | 0.238 |  |
| As | Rarely | Reference |  |  |
|  | Occasionally | -0.006(-0.077,0.064) | 0.86 | 0.362 |
|  | Very Often | 0.037(-0.03,0.105) | 0.281 |  |

Note: Models were adjusted for age, gender, race/ethnicity, education attainment, smoking status, PIR, BMI, consumption of shellfish and fish in the past 30 days, and antihypertensive prescription drug use.

**Supplementary Table 12. Association between urinary levels of 10 metals and the frequency of using salt during cooking or preparing foods after further adjustment for antihypertensive prescription drug use**

| **Urine metals (µg/mg creatinine)** | **Salt used in preparation** | **β (95%CI)** | **p-value** | **p for trend** |
| --- | --- | --- | --- | --- |
| Ba | Never | Reference |  |  |
|  | Rarely | 0.049(-0.041,0.14) | 0.289 | 0.131 |
|  | Occasionally | 0.089(0.006,0.172) | 0.039 |  |
|  | Very Often | 0.071(-0.01,0.151) | 0.089 |  |
| Cd | Never | Reference |  |  |
|  | Rarely | -0.003(-0.067,0.06) | 0.919 | 0.272 |
|  | Occasionally | -0.018(-0.079,0.043) | 0.559 |  |
|  | Very Often | 0.024(-0.042,0.09) | 0.478 |  |
| Co | Never | Reference |  |  |
|  | Rarely | -0.009(-0.071,0.052) | 0.769 | 0.1 |
|  | Occasionally | -0.006(-0.064,0.052) | 0.847 |  |
|  | Very Often | -0.035(-0.091,0.02) | 0.218 |  |
| Cs | Never | Reference |  |  |
|  | Rarely | 0.076(0.029,0.124) | 0.002 | <0.001 |
|  | Occasionally | 0.068(0.023,0.114) | 0.004 |  |
|  | Very Often | 0.106(0.068,0.144) | <0.001 |  |
| Mo | Never | Reference |  |  |
|  | Rarely | -0.018(-0.09,0.053) | 0.618 | 0.017 |
|  | Occasionally | -0.042(-0.109,0.024) | 0.214 |  |
|  | Very Often | -0.062(-0.131,0.006) | 0.079 |  |
| Pb | Never | Reference |  |  |
|  | Rarely | -0.021(-0.087,0.045) | 0.528 | <0.001 |
|  | Occasionally | 0.003(-0.056,0.062) | 0.928 |  |
|  | Very Often | 0.071(0.012,0.13) | 0.02 |  |
| Sb | Never | Reference |  |  |
|  | Rarely | -0.022(-0.095,0.051) | 0.551 | 0.273 |
|  | Occasionally | -0.02(-0.093,0.052) | 0.582 |  |
|  | Very Often | -0.04(-0.112,0.032) | 0.28 |  |
| Tl | Never | Reference |  |  |
|  | Rarely | 0.046(-0.009,0.1) | 0.101 | 0.002 |
|  | Occasionally | 0.056(0.002,0.11) | 0.046 |  |
|  | Very Often | 0.081(0.03,0.132) | 0.002 |  |
| W | Never | Reference |  |  |
|  | Rarely | -0.008(-0.08,0.064) | 0.829 | 0.069 |
|  | Occasionally | -0.005(-0.077,0.068) | 0.902 |  |
|  | Very Often | -0.053(-0.122,0.016) | 0.138 |  |
| As | Never | Reference |  |  |
|  | Rarely | -0.02(-0.118,0.078) | 0.693 | 0.026 |
|  | Occasionally | 0.009(-0.091,0.109) | 0.858 |  |
|  | Very Often | 0.063(-0.041,0.168) | 0.236 |  |

Note: Models were adjusted for age, gender, race/ethnicity, education attainment, smoking status, PIR, BMI, consumption of shellfish and fish in the past 30 days, and antihypertensive prescription drug use.
